# Disparities in Clinical Trial Participation: A Cross-Sectional Survey of Cancer Patients at a Midwest Academic Medical Center

**DOI:** 10.3390/curroncol31090396

**Published:** 2024-09-11

**Authors:** Katie Moreland, Melinda Butsch Kovacic, Shesh Rai, Davendra Sohal

**Affiliations:** 1 Department of Internal Medicine, College of Medicine, University of Cincinnati, Cincinnati, OH 45229, USA; morelake@ucmail.uc.edu; 2 University of Cincinnati Cancer Center, The University of Cincinnati College of Medicine, Cincinnati, OH 45267, USA; butschms@ucmail.uc.edu (M.B.K.); raise@ucmail.uc.edu (S.R.); 3 Department of Rehabilitation, Exercise, and Nutrition Sciences, The University of Cincinnati College of Allied Health Sciences, Cincinnati, OH 45267, USA; 4 Cincinnati Children’s Hospital Medical Center, Department of Pediatrics, College of Medicine, University of Cincinnati, Cincinnati, OH 45229, USA; 5 Department of Environmental and Public Health Sciences, College of Medicine, University of Cincinnati, Cincinnati, OH 45229, USA

**Keywords:** clinical trials, research participation, disparities, diversity, race, cancer

## Abstract

Research conducted on homogenous populations can lead to biased and misleading findings, impeding the development of effective interventions and treatments for diverse populations. Low participation among minority groups further leads to disparities in access to innovative cancer care and treatment outcomes associated with trial participation. To better understand cancer patients’ attitudes and willingness to participate in clinical trials, solid tumor patients attending their clinic visits were invited to complete a survey. The survey included questions on demographics, previous trial participation, and future trial interest. Responses were analyzed with frequency tables and chi-square tests. Of 300 participants, only 96 (32%) were asked to participate in a clinical trial. Of these, 81 (84%) chose to participate and 15 (16%) did not. There were notable differences by race but not gender or education level. Of the 204 who had never been asked to participate, 70% indicated that they would be willing to participate in future, and there was a strong sex–race interaction. Non-White males were the most hesitant group. Of 204, 99% indicated that they would participate to access new treatments, and 57% would participate to contribute to research overall. This study shows that many solid tumor patients undergoing treatment are not offered clinical trials. Racial differences in attitudes toward trial participation are evident. Nonetheless, many patients are willing to participate in trials to access innovative treatments and to support research. Culturally relevant outreach to build trust with minority groups is needed to increase overall participation in clinical trials.

## 1. Introduction

Clinical trials are the mainstay of testing new cancer therapies and have led to many new treatments being approved for use in cancer patients. Clinical trial diversity helps ensure that the treatments approved are effective and safe for their intended populations. However, participation in cancer clinical trials overall remains exceedingly low [1]. Disparities in clinical trial enrollment persist by race, sex, geographic location, and socioeconomic status [2,3,4]. Indeed, more than 40% of cancer clinical trials in the United States (US) do not reflect the cancer incidence rates among diverse racial and ethnic groups, resulting in an over-representation of Whites and an underrepresentation of non-Whites, particularly Black/African American and Hispanic/Latino populations [5]. Underrepresentation of minority groups can lead to sampling bias which minimizes the generalizability of the study’s results [6]. Given the shift in the US population, if nothing is done, the disparities observed will only worsen. In fact, compared to the 2010 US Census, the 2020 Census reported an 88.7% growth in Black/African American and 23% growth in the Hispanic/Latino populations [7]. Others have predicted that by 2045, less than 50% of the US population will be non-Hispanic White [8].

As a clinical trial may be the only remaining viable treatment option for some patients, ensuring equal access to trials [9], reducing cancer disparities and achieving health equity must include equitable access to participation in clinical trials [10]. There are numerous trial-, patient-, and health system-level factors offered for the lack of appropriate representation of Black/African Americans in cancer clinical trials [6]. Eligibility criteria and lengthy enrollment and follow-up requirements are common trial-level factors. System or provider mistrust, lower literacy levels, lower awareness of clinical trials, and financial constraints are examples of patient-level factors. Finally, studies have reported provider implicit bias and attitudes to be critical factors in study enrollment. For example, provider worries about study adherence and retention might keep them from inviting eligible minority patients. This has long been a problem. Indeed, one breast cancer screening trial published nearly 20 years ago indicated that nearly all women reported that their provider had never talked to them about participating in a clinical trial [11]. Unfortunately, minority patients in some health systems continue to have similar experiences.

According to the Cancer Disparities Progress Report by the American Association of Cancer Research (AACR), greater inclusion of Black/African Americans and Hispanic/Latinos among clinical trial participants requires attention to study design with a focus on expanding accrual sites and offering culturally tailored patient education and community outreach, leveraging a more diverse trial workforce including patient navigators, and reducing costs and offering greater financial support for clinical trial participation, particularly given the need for some to travel and take time off work for frequent study visits [12].

Therefore, as a first step to improve overall clinical trial participation within our cancer center, we sought to understand our patients’ attitudes toward and experiences with clinical trials by inviting them to participate in a brief, minimally burdensome survey. We expected that our survey’s results would, in part, help us prioritize strategies to reduce disparities through both our system-level changes and community outreach efforts. Further, we anticipate that the results of this first survey will serve as a baseline for comparison to future survey results to demonstrate the impact of our ongoing and future efforts to improve minority representation within our clinical trial population. Commonly, Clinical Trials Offices (CTOs) are informed by findings reported in the published literature. While local data like these do not typically identify novel targets, they do enable the prioritization of strategies and can buttress efforts to gain investment in those strategies from local and institutional stakeholders. Bringing together Community Outreach and Engagement (COE) groups with CTOs can also increase awareness of the many factors that make clinical trials more representative of the population from which they come, of which many local clinical trialists are unaware.

## 2. Methods

A cross-sectional survey study was carried out at the University Cancer Care Center (UCCC) to evaluate patients’ experiences and attitudes toward clinical trial participation. The survey and related protocol were approved by the University of Cincinnati Institutional Review Board (IRB# 2022-1105).

The survey was administered by a single research assistant (KM) to patients coming for cancer care in various outpatient solid tumor oncology clinics at the UCCC. The survey was a two-page pen and paper survey (Supplementary Text S1), and an intentional effort was made to approach a diverse population of patients by cancer diagnosis clinic, sex, race, time of day, and day of the week. Broadly, patients were asked their name, age, race, and highest grade of education. They were then asked if they had ever been asked to participate in a clinical trial related to their cancer diagnosis. If yes, factors affecting their choice and their future likelihood of participating based on their initial experience were inquired. If they declined to participate initially, reasons for not participating and potential reasons for participating in the future were asked. All questions had prewritten options to choose from, as well as an additional option for open-ended responses. A signed informed consent document was then obtained with consent from each participant to use their survey as part of this study.

All survey responses were entered into a REDCap (Research Electronic Data Capture) database. This database stored all survey answers and allowed the data to be downloaded without any patient identifiers, guaranteeing patient anonymity when analyzing the data. REDCap is a secure, web-based software platform hosted by the Center for Clinical and Translational Science and Training designed to support data capture for research studies, providing (1) an intuitive interface to validate data capture; (2) audit trails for tracking data changes and data exports; (3) the ability to seamlessly download data to common statistical packages; and (4) techniques for data integration with outside sources [13]. Physical copies are secured in a locked cabinet and will continue to be in stored until three years post-publication, where they will then be shredded and destroyed. The database from REDCap was sent only between authors using de-identified data. The database continues to be stored in REDCap and will be kept within REDCap as needed for future research.

This was an exploratory study to understand the perception of participating in future clinical trials. There are many factors that may influence perception. We considered mainly three factors, sex (male and female), race (White, Black, and other), and age (<35, 35–55, and >55) with 18 strata total. A total of 300 consecutive patients from selected clinics were enrolled with an almost equal distribution in sex and age but an unequal distribution in race. The race distribution was expected to be about 60% White, 20% Black, and 20% other. There were many binomial and multinomial responses that were observed. For a comparison between any two groups (such as male versus female), with n1 = 150, n2 = 150 (for sex), and alpha = 0.05, we had 80% power if a binomial response differed by 15% (for example, response to participate in a clinical trial is 65% in males versus 50% in females or vice versa) using a two-sample and a two-sided test for comparing binomial proportions. When comparing the same response in two races (such as White versus Black) with the same design parameters, the detectable difference was 20%. The multinomial responses were exploratory, and power justification was not needed.

Descriptive measures (frequency and percent for discrete variables and mean, standard deviation, median, min, and max for continuous variables) were summarized for the entire cohort and subset of the cohort. The responses were compared between two groups using an appropriate test (t-test for a continuous outcome and a chi-square test for a discrete outcome). Multi-variable logistic regression and multi-variable multinomial regression analyses were used to accommodate for additional covariates [14,15,16,17,18,19]. The results will be declared significant at alpha = 0.05. All analyses were performed in SAS (version 9). We have incorporated all elements of a cross-sectional study per STROBE guidelines.

## 3. Results

From April to August 2023, 390 patients were invited to complete the survey. Of those, 300 patients agreed to and completed the survey (response rate 77%). The median age for the 300 responders was 64 years (range 18 to 93); 168 participants (56%) were women, and 232 (77%) identified as White. Educational attainment (not answered by 18) was up to high school for 153 (54%) and beyond high school for 129 (46%). Educational attainment differed by race; 52% of White participants compared to 24% of non-White participants had education beyond high school (*p* < 0.0001). No major difference was seen in educational attainment by sex, though there was a marginally higher proportion of women who had education beyond high school (50% vs. 41%, NS). Cancer diagnoses included many solid tumors: lung (15%), breast (10%), head/neck (10%), colorectal (8%), pancreatic (8%), ovarian (6%), prostate (6%), endometrial (5%), and others.

To study any effect of the stage of cancer on these results, we divided patients into curable/early-stage cancer vs. incurable/advanced/metastatic cancer since clinical trials often skew toward the latter. In our study population, 114 (38%) patients had curable (stage I, 30; stage II, 34; stage III, 50) cancers and 184 (62%) had incurable cancers (stage data on 2 patients was missing). A race by cancer stage analysis showed no difference—63% White and 59% non-White patients had incurable cancer. Further, for incurable vs. curable cancer patients, 31% vs. 34% were ever offered a trial, and 81% vs. 90% agreed to participate (all comparisons statistically non-significant).

Of 300, only 96 (32%) patients reported ever being asked to participate in a clinical trial. This was disparate by race, as 35% of Whites vs. 21% of non-Whites had ever been asked (*p* = 0.02) (Table 1), but not by sex (32% of men as well as women had ever been asked). Of the 96 who were asked, 81 (84%) consented to participate, while 15 declined. This also differed by race; 88% of Whites vs. 64% of non-Whites agreed to participate (*p* = 0.02) (Table 1). Of note, participation rates did not differ by education level; 83% of those with up to a high school education and 84% of those with beyond high school education participated.

The primary motivations for trial participation among the 81 participants who agreed were to access new treatments (96%) and a desire to contribute to research (75%), with financial incentives being a minor factor (11%). Additionally, 74 of these 81 participants (91%) reported that they would be very likely to participate in future trials, suggesting positive experiences overall.

Regarding reasons for declining participation, among the fifteen patients (ten Whites, five non-Whites) who did so, ten cited aversions to experimental treatments and six raised safety concerns. Importantly, responses were discrepant by race: 0/10 Whites vs. 1/5 non-Whites said they do not trust research/researchers; 3/10 Whites vs. 3/5 non-Whites had safety concerns about clinical trials; and 5/10 Whites vs. 5/5 non-Whites said they did not want experimental treatments.

Among those never asked to participate (204 out of 300), 142 (70%) expressed willingness (very likely) to participate if offered, though racial disparities persisted, with 73% of White and only 59% of non-White participants being willing (*p* = 0.05). Sex and race interactions revealed that 76% of women compared with 62% of men would be very likely to participate if asked in the future, with reluctance more pronounced among non-White men (only 39% were very likely to participate). Table 1 below represents this information in a visual manner.

## 4. Discussion

The goal for NCI-designated cancer centers is to have 15% of patients with cancer enrolled in interventional/therapeutic clinical trials. The most important barrier to research participation reported for oncology patients in this study was being offered enrollment in clinical trials. Indeed, only one-third of our oncology clinic patients were ever offered a trial. That is likely a reflection of a less-than-optimal portfolio of clinical trials at our cancer center.

While we did not see disparities by sex, cancer stage, or educational status, we discovered significant disparities by race. Non-White patients were less likely to be offered a trial. Trial criteria or possibly provider attitudes could be factors influencing trial invitation, although these were not tested in this study. To manage similar provider-level challenges, others have offered training to both their clinical and research staff to help them recognize race and ethnic cultural barriers to participation, minimize the impact of their implicit biases, and improve their cultural humility. In 2022, the American Society of Clinical Oncology (ASCO) and the Association of Community Cancer Centers (ACCC) together published their recommendations to increase equity, diversity, and inclusion (EDI) and address barriers to cancer clinical trial recruitment and participation [20]. Importantly, they too encouraged education and training of personnel involved in designing and offering cancer clinical trials, and further, that the programs should be informed by research participants’ experiences and co-created with patients, community leaders, and other relevant groups. Furthermore, the ASCO-ACCC began promoting the Just ASK™ Training Program [21]. Just ASK™ was adapted from a course co-developed with community representatives at Duke University. It is a freely available, online implicit bias training program intended for all members of the cancer research team.

Provider level challenges withstanding, there is a common misconception that low accrual, especially of racial/ethnic minorities, in clinical trials is due to their disinterest in participation; however, an extensive literature review of 70,000 participants across the US demonstrated that is not generally the case [22]. Notably, we found that most of our study’s survey respondents indicated that they would be open to considering participation in clinical trials if they were invited. While it is speculative, this relative lack of trust can also lead to the confidence and adherence of minority populations to clinical trial treatments and procedures even if they enroll.

Encouragingly, most had enrolled or would enroll in a clinical trial to access new therapies and advance science rather than financial or other logistical reasons. This suggests that there is some level of trust in the scientific aspects of trials. However, we continued to see differences by race. While there were no sex or educational status differences observed, non-White patients (all Black/African Americans) were less likely to join clinical trials when they were offered. Other studies reported similar findings [10,23]. Although sample sizes were small, the survey respondents shared concerns about safety and not wanting to be “experimented on”. Such hesitations are likely due, in part, to historical atrocities, including local incidents, that have engendered mistrust in clinical research and medical institutions [24]. For this reason, it is clear that our institution will need to work harder to earn the trust of minority patients to diminish the observed disparities in clinical trial participation.

To this end, we are working in partnership with our cancer centers’ Community Outreach and Engagement (COE) team. Having such programs is now mandated for all NCI-Designated Cancer Centers [25]. The 2021 Guidelines specify that COE leaders need to work with their CTOs to “facilitate accrual to clinical trials from the catchment area”, as well as develop, implement, monitor, and evaluate strategies that the CTO and its associated researchers implement [26]. These efforts should be informed by the perspectives of the center’s Community Advisory Board. Studies have shown that community-based approaches often raise the participation of underserved, minority populations [22]. Indeed, for the last several years, our COE team has offered an outreach program to increase the “research readiness” of community groups. The outreach leverages a comic-style story co-designed with community representatives to invite non-judgmental discussions about the purpose of research, how participants are protected from harm, and why people of all backgrounds and walks of life are needed to participate. Recently, the story has been adapted to facilitate cancer survivors’ understanding of clinical trials. To support participants’ feelings of safety and trust, the outreach discussions are being facilitated by cancer survivors and not clinical staff.

The strengths of this study include utilizing different cancer clinics and administering surveys at different times during the day. This ensured a wide range of opinions and perspectives from the individuals filling out the surveys. Limitations of this study include the survey being handed out at a single oncology clinic within a larger cancer center. Further, this study is limited to solid tumors, and responses could be affected by varying strengths of clinical trial portfolios in various disease groups. Another limitation is unintentional biases. While we made every attempt to capture patients randomly in different cancer clinics, at different times of day, and during different days of the week, in order to deliberately capture a diverse range of responders, it is possible that we skewed our population toward one group or another.

In summary, our survey study highlights that patients are generally willing to join clinical trials if offered because they want access to new therapies and are eager to participate in scientific progress. However, barriers remain, and racial disparities persist in terms of being offered trials and willingness to join them. Identifying these barriers is an important step forward, and we will focus on reducing them by partnering with our COE and our Community Advisory Board.

## Figures and Tables

**Table 1:** Survey Results for Trial Participation by Race and Gender.

	White (*n* = 232)	Non-White (*n* = 68)	*p*-Value
**Invited to participate in a clinical trial**	82/232 (35%)	14/68 (21%)	0.022
**Chose to participate in a clinical trial**	72/82 (88%)	9/14 (64%)	0.025
**If asked in the future, willing to participate:**	110/150 (73%)	32/54 (59%)	0.054
Women	64/84 (76%)	23/31 (74%)	NS
Men	46/66 (70%)	9/23 (39%)	0.009
**Of those declining, reason:**			
Do not trust research	0/10 (0%)	1/5 (20%)	NS
Safety concerns	3/10 (30%)	3/5 (60%)	NS
Does not want experimental treatments	5/10 (50%)	5/5 (100%)	NS

## Data Availability

The datasets generated and analyzed during this study are available from the corresponding author upon reasonable request.

## Supplementary Materials

The following supporting information can be downloaded at: https://www.mdpi.com/article/10.3390/curroncol31090396/s1, Text S1: University of Cincinnati-Medical Consent to Participate in A Research Study.
