# Supplementary material for: Disparities in Clinical Trial Participation: A Cross-Sectional Survey of Cancer Patients at a Midwest Academic Medical Center
Source: Curr Oncol. 2024 Sep 11;31(9):5367–73. doi: 10.3390/curroncol31090396 (PMC11431411; doi:10.3390/curroncol31090396)
Supplement: Supplementary file 1 [file curroncol-31-00396-s001.zip › curroncol-3185131-supplementary.pdf]

University of CINCINNATI

UNIVERSITY OF CINCINNATI - MEDICAL
CONSENT TO PARTICIPATE IN A RESEARCH STUDY

**STUDY TITLE:**<br><br>**Clinical Trial Participation Behaviors: A Survey of Cancer Patients During Treatment**	
**PRINCIPAL INVESTIGATOR NAME:**<br><br>**Davendra Sohal**	**PHONE NUMBER (24-hour Emergency Contact)**<br><br>513-558-2361
**PARTICIPANT NAME:**	**DATE OF BIRTH:**

INTRODUCTION:

You are being asked to take part in a research study. Please read this paper carefully and ask questions about anything that you do not understand.

WHY IS THIS RESEARCH BEING DONE?

The purpose of this research study is to understand why patients chose to enroll in a clinical trial, whether they would choose to enroll again, and why they chose to enroll at University of Cincinnati. This survey data will help with recruitment for future patients and help with overall knowledge of behaviors of patients enrolling in clinical trials.

WHY HAVE YOU BEEN ASKED TO TAKE PART IN THIS RESEARCH STUDY?

You are being asked to take part in this research study because you are at least 18 years old, and you have been diagnosed with any type of cancer.

You may be included in this study if you:
1. Are at least 18 years old.
2. Must be able to fill out survey in English.
3. Must have received a diagnosis of cancer of any type.
4. Will be receiving or has received treatment with surgery, radiation, chemotherapy, and/or other therapy (e.g., immunotherapy, hormonal etc.) for their cancer diagnosis.
5. Are able to, and provides, informed consent.

The following may exclude you from this study:
1. Adults who lack capacity to consent.
2. Individuals who are not yet adults (infants, children, teenagers).

3. Prisoners
4. Patients who decline primary treatment of surgery, radiation, and/or chemotherapy, and/or other therapy (e.g., immunotherapy, hormonal etc.) for their cancer
5. In the opinion of the investigator the subject is not a good candidate for this study.

WHO IS CONDUCTING THE RESEARCH STUDY?

The study is directed by Dr. Davendra Sohal, a clinician and researcher leading the survey study at the University of Cincinnati Cancer Center.

HOW MANY PEOPLE WILL TAKE PART IN THE RESEARCH STUDY?

Between 300-600 people will take part in this study at the University of Cincinnati Cancer Center, where patients are being seen for treatment.

WHAT WILL YOU BE ASKED TO DO IN THIS RESEARCH STUDY, AND HOW LONG WILL IT TAKE?

You will be asked to fill out a survey regarding questions whether or not you have ever agreed to participate in a clinic trial. It will take about 5 minutes to complete the survey. There is a question at the end of the survey for an optional follow-up call regarding your answers to the survey. That call would take an additional 5-10 minutes. The research will take place within different University of Cincinnati Cancer Clinics. We will also ask for your authorization to go into your medical chart to confirm your answers regarding your cancer diagnosis.

Research will take place at University of Cincinnati and the facilities of the affiliated health systems UC Health, LLC and University of Cincinnati Physicians Company, LLC.

WHAT ARE THE RISKS AND DISCOMFORTS OF THE RESEARCH STUDY?

- It is not expected that you will be exposed to any risk by being in this research study.
- The risk is not expected to be more than you would have in daily life.
- Some questions may make you uncomfortable. You can refuse to answer any questions that you don't want to answer.

ARE THERE BENEFITS TO TAKING PART IN THE RESEARCH STUDY?

If you agree to take part in this research study, there will not be a direct medical benefit to you. We hope the information learned from this research study will benefit other patients with helping clinical trial staff and medical professionals understand patients' thoughts on clinical trial and research studies, which can improve with recruitment and Diversity, Equity, and Inclusion efforts within the University of Cincinnati Cancer Center.

AVAILABILITY OF INFORMATION

You will receive a copy of this signed and dated consent form.

WILL YOU BE PAID TO PARTICIPATE IN THIS RESEARCH STUDY?

There is no cost to being in this study, and you will not be paid to take part in this study.

WHAT ARE YOUR RIGHTS AS A PARTICIPANT?

You may choose either to take part or not to take part in this research study. If you decide to take part, you may decide to leave the study at any time. Leaving the study will not result in any penalty or loss of benefits to you.

If you have questions about the study, you will have a chance to talk to one of the study staff or your regular doctor. Do not sign this form unless you have had the chance to ask questions and have received satisfactory answers.

Nothing in this consent form waives any legal rights you may have, nor does it release the investigator, the institution, or its agents from liability for negligence.

If you do not want to take part in this research study, you may simply not participate.

HOW WILL INFORMATION ABOUT YOU BE KEPT PRIVATE AND CONFIDENTIAL?

Plans that may be used include but are not limited to:

- using a study ID number instead of the participant's name on the research forms
- keeping the master list of names and study ID numbers in a separate location from the research forms
- limiting access to research data to the research team
- not including the participant's name on the typed transcript
- erasing audiotapes as soon as they are transcribed
- keeping research data on a password-protected computer

Your information will be kept in a locked cabinet within Dr. Sohal's office, which will remain locked. It will remain in there during the duration of the analysis of the study, and it is required we keep the information for 3 years after the study is completed, but it will remain in the locked office. After that the information will be destroyed by shredding.

Every effort will be made to maintain the confidentiality of your medical and research records related to this study. The University of Cincinnati, the monitor, the auditor, the Institutional Review Board (IRB), and other regulatory authority(ies*)* will be granted direct access to your original medical and research

records for verification of research study procedures or study data without violating your confidentiality, to the extent permitted by the applicable laws and regulations. By signing this consent form, you or your legally authorized representative are authorizing such access. The data from the study may be published; however, you will not be identified by name. Your identity will remain confidential unless disclosure is required by law.

Authorization to Use and Disclose Health Information

A federal regulation known as the Privacy Rule requires that the researchers get your written permission to use your health care information in this research study. If you sign this form, you are giving your authorization for the use and disclosure of your health information for research purposes. You do not have to give this authorization. If you do not give your authorization you cannot be in the research study. However, if you are being treated as a patient here, you will still be able to receive care.

Who Will Use and Disclose My Health Information? The researchers will use your health information to conduct, review, and determine the results of the study. The researchers may also use your information to prepare reports or publications about the study. Your name will not appear in any report or publication without your permission.

Who Will Receive My Health Information? The following people or groups may receive your health information:

- Researchers who are conducting this study at other study centers
- The study sponsor or its representatives, including companies it hires to provide study-related services
- University of Cincinnati Institutional Review Board (IRB) or an outside external IRB reviewing the study
- Other compliance committees responsible for overseeing the research
- UC Health and UC hospital or clinic employees providing service or care to you
- Federal and State agencies, such as the U.S. Food and Drug Administration (FDA), Department of Health and Human Services (DHHS), the National Institutes of Health (NIH), and other US and non-US government bodies that oversee or review research

Will My Information be Protected by the Privacy Rule After it is Disclosed to Others?
If the groups above share your health information with others, it will no longer be protected by the Privacy Rule.

Will My Authorization Ever Expire? Your authorization will not expire.

May I Take Back My Authorization? You may take back your authorization at any time by writing to the study doctor. If you take back your authorization, you will not be able to stay in this study. If you take back your authorization, the study team will not collect any new health information about you. Information that has already been collected may still be used and given to others. If you withdraw your authorization, no new health information will be collected unless you have a side effect related to the study.

May I Look at My Study Information? You may be able to look at and make copies of your health information collected for this study when the study is completed.

Information that could identify you will be removed from the study data. After removal, the study data could be used for future research studies. The study data could also be given to another researcher for future research studies. This may be done without getting additional permission from you.

WHO DO YOU CALL IF YOU HAVE QUESTIONS OR PROBLEMS?

If you have any questions or concerns about this research study, you should contact the researcher Davendra Sohal at 513-558-2361 or sohalda@uc.edu.

Please call the University of Cincinnati Institutional Review Board at 513-558-5259 (Monday – Friday 8 am to 5 pm) if you:

- Think the research has hurt you.
- Have general questions about giving consent or your rights as a research participant in this research study.
- Have questions, concerns, complaints and/or suggestions about the research.
- Cannot reach the research team or you want to talk to someone else.

To report complaints or concerns to an independent agency in an anonymous and confidential manner, please call the Research Compliance Hotline at 1-800-889-1547.

UNIVERSITY OF CINCINNATI - MEDICAL
CONSENT TO PARTICIPATE IN A RESEARCH STUDY

**STUDY TITLE:**<br><br>**Clinical Trial Participation Behaviors: A Survey of Cancer Patients During Treatment**	
**PRINCIPAL INVESTIGATOR NAME:**<br><br>**Davendra Sohal**	**PHONE NUMBER (24-hour Emergency Contact)**<br><br>513-558-2361
**PARTICIPANT NAME:**	**DATE OF BIRTH:**

I have read or someone has read to me, this Informed Consent Document which describes the purpose and nature of this research. I have had time to review this information and have been encouraged to ask questions. If I do not participate or if I discontinue my participation, I will not lose any benefits or any legal rights. My participation in this research is completely voluntary. I have received (or will receive) a copy of this signed and dated form for my records and future reference. I have been given the information about the use and disclosure of my health information for this research study.

PERSON OBTAINING CONSENT
I have read this form to the participant and/or the participant has read this form. An explanation of the research was given and questions from the participant were solicited and answered to the participant's satisfaction. In my judgment, the participant has demonstrated comprehension of the information.

Signature and Title of Person Obtaining Consent and Identification of Role in the Study — Date

Participant — Date
